# Escala predictiva de fallo renal agudo en sepsis (ARMO)

**DOI:** 10.15446/rsap.V25n2.105124

**Published:** 2023-03-01

**Authors:** Christian L. Mora-Coello, Andrea C. Armendáriz-Carvajal, Jorge L. Vélez-Paez

**Affiliations:** 1 CM: MD. Esp. Medicina Crítica y Terapia Intensiva. Hospital General Monte Sinaí. Guayaquil, Ecuador. dr.christianmora@gmail.com Medicina Crítica y Terapia Intensiva Hospital General Monte Sinaí Guayaquil Ecuador; 2 AA: MD. Esp. Medicina Crítica y Terapia Intensiva. Hospital General Pablo Arturo Suárez. Quito, Ecuador. andreaarmendarizmd@gmail.com Medicina Crítica y Terapia Intensiva Hospital General Pablo Arturo Suárez Quito Ecuador; 3 JV: MD. Esp. Medicina Crítica y Terapia Intensiva. Ms. Investigación Clínica. Hospital General Pablo Arturo Suárez. Quito, Ecuador. jorgeluisvelezl3@hotmail.com Investigación Clínica Hospital General Pablo Arturo Suárez Quito Ecuador

**Keywords:** Injuria renal aguda, sepsis, AKI score *(fuente: DeCS, BIREME)*, Acute kidney injury, sepsis, AKI score *(source: MeSH, NLM)*

## Abstract

**Objetivo:**

Definir la utilidad predictiva de la escala adaptada de Injuria Renal Aguda (ARMO) en los pacientes sépticos en las Unidades de Cuidados Intensivos de Quito durante el período 2020 a 2021.

**Materiales y Métodos:**

Estudio observacional, descriptivo, ambispectivo, multicéntrico de pacientes sépticos en dos Unidades de Cuidados Intensivos de la ciudad de Quito, Ecuador, con una muestra de 200 pacientes, y datos obtenidos en las primeras 72 horas de ingreso, que incluyeron variables demográficas y clínicas, medidas terapéuticas y de intervención, sometidas a análisis multivariado con regresión logística.

**Resultados:**

Se analizaron 200 pacientes, con una mediana de edad 57 años. El 41 % (82) presentaron falla renal y el 40,96 % correspondieron a estadio KDIGO 3. El 11,5 % de los pacientes con injuria renal requirió terapia sustitutiva renal. Tras el análisis multivariado se determinó que: la TFG ≤84 ml/min/1,73m2, lactato sérico ≥2,5 mmol/l, SOFA ≥10 puntos y gasto urinario ≤0,6 ml/kg/h son predictores de falla renal. A partir de ello, se plantea una nueva escala predictiva de falla renal aguda, score ARMO, con una curva ROC de 0,836 (IC 95 %, 0,781-0,890) con un punto de corte de 8 puntos.

**Conclusión:**

La escala adaptada de Injuria Renal Aguda (ARMO) es una herramienta con alta capacidad discriminativa en los pacientes críticos sépticos.

## Abstract (en)

**Objective:**

To define the predictive utility of the adapted scale of Acute Renal Injury (ARMO) in septic patients in the Intensive Care Units of two hospitals in Quito during the period 2020 to 2021.

**Materials and Methods:**

Observational, descriptive, ambispective, multicenter study of septic patients in two Intensive Care Units in the city of Quito, Ecuador, with a sample of 197 patients, with data within the first 72 hours of admission, analysis of demographic and clinical variables, therapeutic and intervention measures, values of prognostic scales and multivariate analysis with logistic regression.

**Results:**

200 patients were analyzed, with a median age of 57 years, 41 % (82) presented kidney failure, and 40.96 % corresponded to KDIGO stage 3. 11.5 % of patients with kidney injury required renal replacement therapy. After multivariate analysis, it was determined that: GFR ≤84 ml/min/1.73m2, serum lactate ≥2.5 mmol/l, SOFA ≥10 points and urinary output ≤0.6 ml/Kg/h are predictors of renal failure. Based on this, a new predictive scale for acute renal failure, ARMO score, with a ROC curve of 0.836 (95 % CI, 0.781-0.890) with a cut-off point of 8 points, is proposed.

**Conclusion:**

The adapted scale of Acute Renal Injury (ARMO) shows a high discriminative capacity.

El fallo renal agudo se define como el deterioro súbito de la función renal con acumulación de productos nitrogenados (BUN, creatinina) y otros desechos, los cuales en condiciones fisiológicas son excretados por los ríñones 1. Fue descrita inicialmente en 1802 en un paciente anúrico y definida como ischuria renalis por W. Heberden. A inicios del siglo XX fue asociada a intoxicaciones, quemaduras, estados postquirúrgicos y postraumáticos. En 1908 fue descrita como Enfermedad Aguda de Bright por William Osler 2.

El término fallo renal agudo (FRA) fue descrito por William MacNider en un caso de intoxicación aguda por mercurio 3 y desde 2004 se convirtió en el término más empleado. Posteriormente, se han llevado a cabo varias redefiniciones en consensos, como RIFLE y después el grupo AKIN. En el año 2012, el Grupo de Trabajo KDIGO propuso criterios unificados basados en RIFLE y AKIN 4.

La sepsis es una patología común en las Unidades de Cuidados Críticos. Durante varios años, se la relacionó estrechamente con el síndrome de respuesta inflamatoria sistémica. Su definición ha sido un tema de discusión científica a lo largo del tiempo. Actualmente, se define como "la respuesta desregulada del organismo ante una infección" y se evalúa utilizando la escala SOFA, considerando un valor igual o mayor a 2 puntos 5.

Una disfunción añadida en la sepsis es la lesión renal, la cual se caracteriza por su etiología multifactorial. Se cree que los mecanismos iniciales incluyen la hipoperfusión y vasoconstricción renal, así como la hipovolemia relativa debido a la vasoplejía y la fuga capilar como respuesta a la cascada inflamatoria sostenida. Además, los pacientes sépticos pueden experimentar hipoxia citopática, un estado en el cual, a pesar de mejorar el suministro de oxígeno, la maquinaria enzimática y mitocondrial de las células se ha visto severamente comprometida por la infección 6.

Varios investigadores han colaborado en la identificación de factores directamente relacionados con la mortalidad y el desarrollo de lesión renal. Se ha observado una asociación positiva entre variables como la diabetes, el daño hepático crónico, la insuficiencia cardíaca, la edad avanzada y el sexo masculino. Además, se ha encontrado que el uso de inhibidores de la enzima convertidora de angiotensina (IECAS) y diuréticos puede tener repercusiones en los riñones. Por otro lado, valores altos en las escalas SOFA y APACHE II, así como el uso de ventilación mecánica y vasopresores, han mostrado resultados divergentes en varios estudios, principalmente observacionales 7.

En la actualidad, varios biomarcadores se emplean para identificar con antelación la injuria renal aguda, lo que los convierte en elementos pronósticos, incluso para terapia sustitutiva renal y progresión al daño renal crónico. Entre estos biomarcadores se encuentran TIMP-2 e IGFBP-7, que son marcadores de detección en la fase G1 del ciclo celular. Tras una lesión renal, estos biomarcadores activan los mecanismos de división y proliferación celular para reparar el epitelio tubular. Estos fenómenos pueden ocurrir hasta 48 horas antes de que se observen cambios en los niveles séricos de creatinina 8.

La disponibilidad y acceso a instrumentos y tecnología de detección precoz de lesión renal aguda no son universales en todas las instituciones sanitarias, por lo que es importante desarrollar herramientas clínicas que permitan predecir con alta precisión este tipo de fallo renal. Es por eso que se han propuesto múltiples escalas predictivas que consideran características agudas y crónicas, las cuales han sido desarrolladas y validadas tanto en pacientes sépticos como no sépticos.

Se desarrolló una escala predictiva de fallo renal a través de un estudio prospectivo y multivariado. En este estudio, se identificaron tendencias en pacientes sépticos que mostraban factores de riesgo, como pH <7.30, exposición a nefrotoxinas, enfermedad renal crónica, hepatopatía crónica, insuficiencia cardíaca congestiva, hipertensión, aterosclerosis, ventilación mecánica y anemia. Estos factores se agruparon bajo un modelo de predicción de riesgo que demostró un área bajo la curva (AUROC) de 0.81 9. En Italia, durante el año 2019, se desarrolló el Quick AKI Score, el cual incorporó variables específicas, cortas y con resultados prometedores con un AUROC de 0.79 10; si bien incluye a pacientes críticamente enfermos, no distingue entre sépticos y no sépticos.

## MATERIALES Y MÉTODOS

### Diseño y tipo de estudio

Estudio de tipo observacional, descriptivo, ambispectivo, multicéntrico, en pacientes sépticos en las Unidades de Cuidados Intensivos de dos hospitales de Quito, Ecuador, con diagnóstico de sepsis. El estudio se llevó a cabo con el consentimiento y la aprobación de los Comités de Bioética de cada una de las unidades de salud involucradas en el estudio. El período de estudio abarcó desde 2020 hasta 2021.

### Población de estudio

Para la selección de la muestra, se consideraron todos los pacientes ingresados en las Unidades de Terapia Intensiva de los hospitales General Docente de Calderón y Pablo Arturo Suárez, que contaban con el diagnóstico de sepsis y cumplían con los criterios de inclusión. Los pacientes seleccionados fueron aquellos hospitalizados en el período comprendido entre enero de 2020 y septiembre de 2021.

Criterios de inclusión y exclusión: para la inclusión, se consideraron hombres y mujeres mayores de 18 años de edad con diagnóstico de sepsis según los criterios de SEPSIS-3, que ingresaron a la Unidad de Cuidados Intensivos durante el período 2020 y 2021. Los criterios de exclusión incluyeron hombres y mujeres menores de 18 años de edad, mujeres embarazadas y pacientes con las siguientes condiciones: enfermedades neoplásicas, insuficiencia renal crónica o reagudizada, en terapia de sustitución renal, cirrosis hepática, insuficiencia cardíaca en etapas III y IY según la clasificación NYHA, en limitación del esfuerzo terapéutico, infección por YIH/SIDA y pacientes que no requirieron ventilación mecánica invasiva.

Aleatorización: en la fase retrospectiva se empleó el método de muestreo probabilístico aleatorio simple y en la fase prospectiva se aplicó el método de muestreo no probabilístico por conveniencia.

Recolección de datos: se utilizó un formulario para recopilar datos de los historiales clínicos, incluyendo información demográfica, clínica y evaluativa, parámetros hemodinámicos, marcadores renales, ventilatorios y datos de laboratorio. También se registraron datos relacionados con el tratamiento y el resultado del paciente. Una vez obtenidos los datos, se introdujeron y codificaron en el software estadístico SPSS Stadistics 25.0.

### Análisis de datos

Las variables cualitativas, tanto nominales como ordinales, se analizaron mediante frecuencias absolutas y relativas, y se presentaron en tablas de contingencia para describir las características clínicas de los pacientes incluidos en el estudio. Por otro lado, las variables cuantitativas, tanto discretas como continuas, se analizaron utilizando medidas de tendencia central, como la mediana y la media, y se evaluó la dispersión de los datos mediante la desviación estándar.

Con la finalidad de establecer la capacidad predictiva de la escala, se ejecutó un análisis con curva ROC en función de los componentes del score q-AKI 10. Solo aquellos cuya área bajo la curva fue representativa se consideraron para la asignación del puntaje previa ejecución de la regresión logística. Se determinaron nuevos componentes como parte de una escala predictiva mediante el cálculo de la curva ROC, y su interpretación se realizó utilizando el método de Swets. Los resultados obtenidos se expresaron en forma de Odds ratio con un intervalo de confianza del 95 %. En todos los casos, se estableció un valor de p inferior a 0.05 como nivel de significancia estadística.

### Aspectos bioéticos

Este estudio se adhiere a los principios establecidos en la Declaración de Helsinki de 2008, que garantiza la protección de la confidencialidad de la información obtenida de los registros médicos de los individuos. Los datos de identificación personal de los pacientes fueron omitidos para preservar su privacidad. Se obtuvieron las autorizaciones correspondientes de los departamentos de docencia e investigación de los hospitales: Hospital General Docente de Calderón - Hospital Pablo Arturo Suárez, así como del Comité de Bioética de la Pontificia Universidad Católica del Ecuador.

## RESULTADOS

Se analizaron los datos de 200 pacientes críticos con sepsis. La mediana de edad fue de 57 años, con un 53,5 % de pacientes de sexo femenino y un 46,5 % de pacientes de sexo masculino. Las comorbilidades más comunes fueron hipertensión arterial en un 25 % de los pacientes y diabetes mellitus en un 23 % de los pacientes. La mediana del índice de Charlson fue de 2 puntos en los pacientes con injuria renal. En cuanto al sitio de infección, se observó que la infección pulmonar fue la más recurrente, presentándose en un 46,5 % de los casos. Le siguieron las infecciones abdominales en un 33 % de los casos, las infecciones del tracto urinario en un 16 % de los casos, las infecciones del sistema nervioso central en un 2,5 % de los casos y las infecciones de partes blandas en un 2 % de los casos.

El 41 % (82) presentaron falla renal aguda, según la clasificación KDIGO, distribuidos en los siguientes estadios: 32,53 % en el estadio KDIGO 1, 26,51 % en el estadio KDIGO 2 y 40,96 % en el estadio KDIGO 3. El 11,50 % de los pacientes requirió terapia sustitutiva renal.

La estancia hospitalaria promedio fue de seis días. La tasa de mortalidad global fue del 20 %. Sin embargo, al analizar los subgrupos, se observó que la mortalidad fue del 13,56 % en los pacientes sépticos sin falla renal, mientras que en los pacientes sépticos con falla renal fue del 29,27 %, lo que representa una diferencia de mortalidad del 15,71 %.

Análisis bivariado: se encontró que las variables índice de masa corporal (IMC), presión positiva al final de la espiración (PEEP), PaO_2_/FiO_2_ (índice de Kirby) y volumen tidal (YT) no mostraron significancia estadística. Sin embargo, el balance hídrico, el gasto urinario, la tasa de filtrado glomerular, el lactato sérico y el score de SOFA mostraron una significancia estadística con un valor de p<0,001 (Tabla 1).

**Figura 1:**
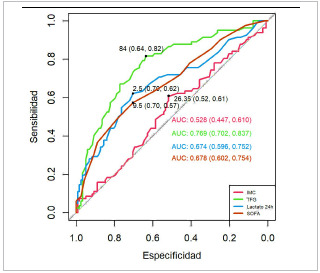
Curvas ROC del IMC, TFG, lactato a las 24 horas y SOFA para predecir falla renal aguda

Análisis multivariado y regresión logística: después del análisis bivariado, se llevó a cabo un análisis de regresión logística y se construyeron curvas ROC para cada parámetro significativo. Los resultados revelaron que el balance hídrico, el gasto urinario, la tasa de filtrado glomerular, el lactato sérico y el puntaje de SOFA son predictores de falla renal aguda. Área bajo la curva: (Figura 1 y 2).

**Tabla 1:** Comparación de los parámetros propuestos en la escala modificada de Injuria Renal Aguda (Q-AKI score) con la ausencia y presencia de falla renal aguda

Parámetros	Falla renal		p-valor
	Ausente	Presente	
IMC (mediana (IQR)) Kg/m^2^	26 (24-29,85)	27 (24-29,85)	0,495
Balance hídrico (mediana (IQR)) ml	-101 (-641-414,5)	497 (-283-1061,25)	<0,001^*^
Gasto urinario (mediana (IQR)) ml/Kg/h	1 (0,8-1,3)	0,6 (0,4-1,3)	<0,001^*^
TFG^+^ (mediana (IQR)) ml/min/1,73m^2^	94 (77-109)	67 (53-80,25)	<0,001^*^
Volumen tidal (mediana (IQR)) ml/kg	468 (397-511,5)	454 (399-512,25)	0,848
PEEP (mediana (IQR)) cmH_2_O	5 (5-8,5)	5 (5-8,25)	0,802
PaO_2_/FiO_2_ (mediana (IQR)) mmHg	159 (108-208,5)	161 (106-208,5)	0,792
Lactato (mediana (IQR)) mmol/l	2 (2-2,7)	3 (2-4,95)	<0,001^*^
SOFA* (mediana (IQR))	8 (5-10)	10 (7-13)	<0,001^*^

Fuente: Hospitales participantes. Nota: IQR= Rango Intercuartílico; ^*^ diferencias significativas prueba Mann Whitney Abreviaturas: TFGt Tasa de Filtrado Glomerular, SOFA^*^ Escala SOFA/Disfunción Multiorgánica.

- Balance hídrico: AUROC de 0,648 (IC-95 %, 0,5660,729) con punto de corte de 49 mililitros, sensibilidad del 68 % y especificidad del 62 %.
- Gasto urinario: AUROC de 0,651 (IC-95 %, 0,5670,734), con un punto de corte de 0,6 ml/Kg/h, sensibilidad del 51 % y especificidad del 84 %.
- Tasa de filtrado glomerular (TFG): AUROC de 0,769 (IC-95 %, 0,702-0,837), punto de corte de 84 ml/ min/1.73m2, sensibilidad del 82 % y especificidad del 62 %.
- Lactato sérico a las 24 horas: AUROC de 0,674 (IC-95 %, 0,596-0,752), punto de corte de 2,5 mmol/l, sensibilidad del 62 % y especificidad del 70 %.
- Puntaje de SOFA: AUROC de 0,678 (IC-95 %, 0,6020,754), punto de corte de 10 puntos, sensibilidad del 57 % y especificidad del 70 %.

Fuente: Hospitales participantes. Mora Coello C., Armendáriz Carvajal A. Escala predictiva de fallo renal agudo en sepsis (ARMO), 2022.

**Figura 2:**
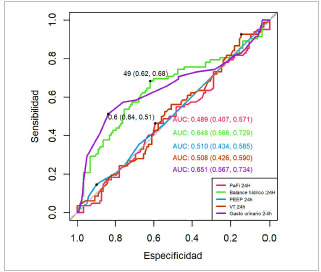
Curvas ROC de: PaO2/FiO2, balance hídrico, PEEP, volumen tidal y gasto urinario para predecir falla renal aguda

Fuente: Hospitales participantes Mora Coello C., Armendáriz Carvajal A. Escala predictiva de fallo renal agudo en sepsis (ARMO), 2022.

En un primer modelo de regresión logística, la TFG y el gasto urinario fueron predictores de falla renal con p-valores <0,001. Se ha demostrado que un valor de TFG ≤84 ml/min/1,73m^2^ tiene 6,46 veces más probabilidades de desarrollar falla renal aguda, mientras que un gasto urinario ≤0,6 ml/kg/h confiere 4,49 veces más probabilidades de falla renal. El score SOFA y el lactato presentaron valores p de 0,062 y 0,067, respectivamente, lo que indica una tendencia hacia la significancia estadística. Por otro lado, el balance hídrico no mostró una asociación significativa, con un valor p de 0,253.

Se ajustó un nuevo modelo de regresión logística excluyendo el balance hídrico con base en lo expuesto. Los resultados mostraron que una TFG ≤84 ml/min/1,73m2, lactato sérico ≥2,5 mmol/l y gasto urinario ≤0,6 ml/ Kg/h son predictores de falla renal con valores p<0,001 y 0,043, respectivamente. Estos parámetros presentaron 7,15, 2,07 y 4,81 veces más probabilidad de desarrollar falla renal aguda, respectivamente. Además, un puntaje SOFA ≥10 se asoció con un riesgo 2,03 veces mayor de desarrollar injuria renal aguda, aunque el valor p correspondiente fue de 0,056, indicando una tendencia hacia la significancia estadística.

Se consideraron los valores de Odds Ratio del análisis multivariante como los puntajes a tener en cuenta en la escala propuesta. Si algún valor difiere del punto establecido, se deberá asignar un valor de cero (0), lo que resulta en un rango de puntuación mínima de 0 puntos y máxima de 16 puntos (Tabla 2).

**Tabla 2:** Puntaje para los posibles factores de riesgo de falla renal

Escala adaptada de injuria renal aguda	Puntaje
SOFA * ≥10 puntos	2 puntos
TFG+ ≤84 ml/min/1,73m^2^	7 puntos
Lactato sérico ≥2,5 mmol/l	2 puntos
Gasto urinario ≤0,6 ml/Kg/h	5 puntos
Total	16 puntos

Fuente: Hospitales participantes. Abreviatura: SOFAJ Esccala SOFA/Disfunción Multiorgánica, TFGf Tasa de Filtrado Glomerular. Mora Coello C., Armendáriz Carvajal A. Escala predictiva de fallo renal agudo en sepsis (ARMO), 2022.

**Figura 3:**
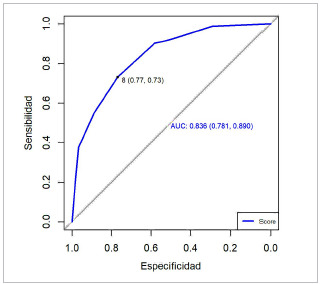
Curvas ROC del score propuesto para predecir falla renal aguda

Basados en los análisis realizados, se elaboró la curva ROC para la falla renal aguda de la escala propuesta, obteniéndose un valor de 0,836 (IC 95 %, 0,781-0,890). Esto indica que el score propuesto es un buen predictor de la injuria renal aguda, con un punto de corte de 8 puntos, una sensibilidad del 73 % y una especificidad del 78 %. Por lo tanto, un valor ≥ 8 puntos, con un valor p<0,001, muestra que hay 9 veces más probabilidad de desarrollar injuria renal aguda entre los pacientes sépticos (Figura 3 y Tabla 3).

Fuente: Hospitales participantes. Mora Coello C., Armendáriz Carvajal A. Escala predictiva de fallo renal agudo en sepsis (ARMO), 2022.

**Tabla 3:** Relación del score propuesto para predecir falla renal

Variables	B	Wald	p-valor	OR	IC-OR 95 %	
					Inferior	Superior
Score ≥8 puntos	2,21	44,18	<0,001*	9,09**	4,74	17,43

Fuente: Hospitales participantes. Mora Coello C., Armendáriz Carvajal A. Escala predictiva de fallo renal agudo en sepsis (ARMO), 2022.

Nota: Valores basados en la prueba chi-cuadrado; * variable significativa p-valor<0,05, ** OR= Odds ratio significativo; basada en Regresión Logística.

## DISCUSIÓN

La incidencia de injuria renal aguda en sepsis varía ampliamente alrededor del mundo, Bagshaw *et al.*^11^ analizaron más de 120.000 pacientes de UCI australianas y encontraron una incidencia de 11.7 %; a diferencia del estudio de Wang *et al.*^12^, quienes estudiaron datos de 19 579 pacientes durante un período de 12 años de UCI en Beijing, China con 59.2 % de incidencia y 55 % de mortalidad. En nuestro estudio, el 41 % de los pacientes sépticos desarrollaron falla renal aguda, un valor más cercano a los datos referidos por Wang *et al.,* con un corte etáreo de 57 años y mayor frecuencia entre las mujeres 53,5 %.

En la cohorte de Ferrari *et al.*^10^, el 37,9 % de pacientes críticos desarrollaron falla renal aguda, el 60,3% en estadio 1. Nuestro estudio revela datos trascendentales; casi la mitad de los pacientes sépticos desarrollaron injuria renal y el estadio KDIGO 3 fue el predominante.

El uso de norepinefrina y su relación con la IRA presenta resultados contradictorios. Se ha observado que una presión arterial media (TAM) inferior a 73 mmHg y una dosis de norepinefrina mayor a 0,19 mcg/kg/min son factores independientes asociados a la progresión de la IRA 13. El ensayo YANISH determinó que en el grupo de pacientes que recibió norepinefrina, la incidencia de IRA fue mayor en comparación con el grupo que recibió vasopresina. Además, se observó una mayor necesidad de terapia de reemplazo renal en el grupo de norepinefrina, con una diferencia porcentual de 9.9 %. Sin embargo, es importante destacar que este estudio no identificó una asociación de riesgo entre el uso de norepinefrina y la IRA 14, algo que tampoco pudimos demostrar.

La sobrecarga hídrica se relaciona directamente con la IRA al ocasionar disfunción endotelial, que a su vez causa daño al glicocálix. Esto puede llevar a la aparición de diferentes efectos secundarios, como edema pericapilar, fuga capilar, congestión renal y edema intersticial 15,16. Vélez *et al.*^16^, definen al glicocálix como "el regulador por trascendencia de la homeostasis a nivel vascular", también afectado en sepsis por la sobreexpresión de factores de adhesión leucocitaria al endotelio. Aunque la resucitación con fluidos es una medida fundamental en el manejo inicial del paciente séptico, sus efectos deletéreos pueden empeorar la disfunción renal 17.

Durante años se ha demostrado que la sobrecarga de fluidos se asocia con un peor pronóstico en la UCI. Un estudio prospectivo reciente ha demostrado que un balance hídrico superior a 3,4 litros y un gasto urinario menor a 0,85 litros por día son factores independientes asociados con el desarrollo de IRA y la mortalidad 18. Sin embargo, no pudimos determinar asociación positiva entre un balance hídrico positivo y la lesión renal, lo que contrasta con Nogueira *et al.*^18^, quienes mostraron que un incremento de 100 ml de balance hídrico se asocia con un ascenso del 4 % de riesgo de IRA, y que el balance positivo hacia el cuarto día es un marcador temprano de fallo.

El empleo previo de furosemida en pacientes críticamente enfermos exhibe resultados contradictorios. Krzych *et al.*^19^ evaluaron el impacto de la furosemida a una dosis < o > a 160 mg/día, sobre la fatalidad y necesidad de terapia de sustitución renal y no encontraron relación estadística. En nuestro estudio tampoco pudimos demostrar asociación causal.

Las comorbilidades asociadas a IRA en sepsis incluyen hipertensión arterial, diabetes mellitus, enfermedad cardiovascular, enfermedad coronaria y enfermedad hepática. Además, la presencia de infección abdominal y otras variables clínicas como el choque y la ventilación mecánica se han asociado positivamente con el desarrollo de IRA 20. En nuestro análisis de resultados sugerimos que ni la hipertensión arterial, ni el índice de Charlson ≥2, ni tampoco el sitio de infección tienen asociación significativa.

A la luz de la evidencia, es claro que la IRA en sepsis tiene una amplia variedad de factores precipitantes, lo que dificulta y complica el establecimiento de una estrategia única de intervención y prevención. Basados en ello, se han diseñado varios modelos probabilísticos, Zhou *et al.*^21^ crearon un modelo probabilístico en pacientes sépticos con 16 variables con un AUROC 0.861. Por otro lado, Yue *et al.* construyeron un modelo de 11 variables: comorbilidades y datos paraclínicos (hemoglobina, albúmina, otros) con un AUROC de 0.76 22. Es claro que los modelos mencionados y otros tantos pese a tener una buena capacidad predictiva, incluyen numerosos factores que a la hora de aplicarlos al pie de cama resulten poco prácticos.

Este estudio se basó en la escala desarrollada por Ferrari *et al.,* que es un modelo fácil y conciso. La escala incluyó cinco elementos: TFG <90 ml/min/1,73m2, SOFACV ≥2, presencia de obesidad, lactato ≥2 mmol/L y un valor ≥0,3 ng/ml del producto de los factores TIMP-2IGFBP7. Sin embargo, es importante mencionar que el último elemento, el biomarcador TIMP-2IGFBP7, no está disponible en las UCI de Ecuador ni en la mayoría de los países en vías de desarrollo 10.

Por lo tanto, proponemos un nuevo modelo adaptado de la escala previamente mencionada, el cual puede ser aplicable en cualquier unidad crítica a nivel mundial. Este nuevo modelo considera cuatro variables fundamentales: SOFA ≥10 puntos, TFG ≤84 ml/min/1.73m2, lactato sérico ≥2.5 mmol/L y gasto urinario ≤0.6 ml/Kg/h. Estas variables hacen que esta herramienta sea de aplicación sencilla, precisa y rápida, y presenta una curva ROC de 0.83.

Como limitaciones de nuestro estudio, debemos destacar lo siguiente: en primer lugar, se trata de un estudio observacional que es ambispectivo, lo que implica que parte de la información recolectada corresponde a una cohorte retrospectiva, lo que dificulta la generalización de los resultados obtenidos. En segundo lugar, no se incluyeron en el análisis variables adicionales como otras condiciones crónicas o marcadores paraclínicos, lo que podría haber aportado información adicional relevante. Por último, debido a la falta de disponibilidad de biomarcadores como el TIMP-2*IGFBP7 en nuestro país, no fue posible analizar la capacidad predictiva de este elemento en pacientes con sepsis.

La fortaleza de este trabajo radica en que se han estudiado factores específicos dentro de una condición crítica, como es la sepsis. Esto hace que esta escala sea un modelo novedoso que permitirá predecir el desarrollo de injuria renal en la etapa aguda del paciente crítico. Se trata de un instrumento clínico que puede ser utilizado de manera rutinaria con datos accesibles y fáciles de obtener dentro de las primeras 24 horas de admisión a Cuidados Intensivos. El objetivo es lograr un diagnóstico más temprano y aplicar intervenciones adecuadas y oportunas.

La prevalencia de disfunción renal aguda en pacientes sépticos es elevada y está influenciada por factores como la edad mayor de 64 años, hipertensión arterial y un índice de Charlson igual o mayor a 2 puntos. Su mortalidad se duplica en asociación con sepsis. Cuatro de cada diez pacientes sépticos desarrollarán falla renal aguda, y la mayoría de ellos alcanzarán el estadio KDIGO 3. Además, uno de cada diez pacientes requerirá terapia de soporte renal. Parece que la escala ARMO sería un método útil para predecir el desarrollo de falla renal aguda en pacientes sépticos en las UCI, ya que muestra una alta capacidad discriminativa ♦
